# Myokines derived from contracting skeletal muscle suppress anabolism in MCF7 breast cancer cells by inhibiting mTOR

**DOI:** 10.3389/fphys.2022.1033585

**Published:** 2022-10-21

**Authors:** Amanda R. Davis, Chelsea G. Goodenough, Kim C. Westerlind, Robert Strange, John W. Deaver, Patrick J. Ryan, Steven E. Riechman, James D. Fluckey

**Affiliations:** ^1^ Texas A&M University, School of Education and Human Development, College Station, TX, United States; ^2^ University of Colorado Health Sciences Center, Denver, CO, United States

**Keywords:** protein synthesis, tumor suppression, cell culture, electrical stimulation, myokines

## Abstract

**Summary:** There is strong evidence that physical activity has a profound protective effect against multiple types of cancer. Here, we show that this effect may be mediated by factors released from skeletal muscle during simulated exercise, *in situ*, which suppress canonical anabolic signaling in breast cancer. We report attenuated growth of MCF7 breast cancer cells in the presence of a rodent-derived exercise conditioned perfusate, independent of prior exercise training. This reduction was concomitant with increased levels of DEPTOR protein and reduced mTOR activity.

## Introduction

Cancer is the second leading cause of death in the United States, coming in a narrow second only to heart disease (National Vital Statistics Reports, Vol. 68, No. 9, 24 June 2019, 2019). Of the primary malignancies, cancers of the breast are the most common type of tumor in women, with the American Cancer Society reporting 276,480 new cases and over 42,170 deaths in 2020 alone (Siegel et al., 2020). While great advances in radiological and pharmacological treatments for breast cancer have been made, there has been growing recognition of the modifiable risk factors that lead to tumor development, with one of the chief beneficial lifestyle choices being regular physical activity.

Regular exercise as a key player in reduction of tumor growth has long been noted (Shephard, 1993; Thune et al., 1997; Lee, 2003; Westerlind, 2003), with numerous studies demonstrating that habitual physical activity decreases the risk of developing breast cancer (Bernstein et al., 1994, 2005; Dethlefsen et al., 2017) and improves survival after cancer diagnoses (Holmes et al., 2005; Chen et al., 2011; Hayes et al., 2018). Much of the prior literature on this topic has ascribed these benefits to improvements in systemic factors such as improved body composition (Brown et al., 2016; Iwase et al., 2021), reductions in inflammatory markers (Murphy et al., 2011; Meneses-Echávez et al., 2016) or stabilization of circulating hormones (Friedenreich et al., 2010; de Roon et al., 2018; Chang et al., 2020). While these events are undoubtedly important to overall homeostasis and health, the identification of myokines, pharmacologically active compounds released from skeletal muscle (Pedersen et al., 2007), has led to a new appreciation of the role of muscle in mediating the metabolism and function of numerous cells and tissues within the body *via* inter-organ cross-talk. Since the seminal discovery of these signaling molecules (Febbraio et al., 2004), much work has been done to identify the specific myokines released from muscle (Aoi et al., 2013; Raschke et al., 2013; Colaianni et al., 2015), and investigate potential cross-talk between muscle and other organs *via* the myokine(s) (Severinsen and Pedersen, 2020; Chen et al., 2021). As a result of this research, there is convincing evidence to suggest that during exercise, contracting skeletal muscle functions as an endocrine-like organ that can regulate the activity of other tissues—including malignant tumors (Pedersen, 2011; Hoffmann and Weigert, 2017; Hojman et al., 2018; Schwappacher et al., 2021) - by releasing pharmacologically active signaling molecules into circulation. Thus, the present study sought to investigate the effect of myokines derived from contracting skeletal muscle on anabolism in breast cancer cells, and determine the mechanism by which these compounds exert their influence.

Since it is identification several decades ago, the serine/threonine kinase mTOR has been a target of interest in both the stimulation and suppression of cell growth. mTOR receives inputs from a variety of sources, including upstream signaling from the PI3K/AKT pathway, nutrients, and energy availability, regulating anabolic processes involving cap-dependent mRNA translation, lipid biosynthesis, and, depending on its binding partners, the inhibition of autophagy (Saxton and Sabatini, 2017). Since mTOR is at a nexus of cellular signaling networks that control the anabolic and catabolic processes inside every cell in the body, it makes this kinase an attractive target for therapies against diseases of altered metabolism such as cancer. Years of both basic and clinical scientific inquiry have identified the mTOR kinase as being highly active in numerous tumors (Zoncu et al., 2011; Mossmann et al., 2018), including those of the breast (Paplomata and O’Regan, 2014), underlying the out-of-control growth characteristic of rapidly dividing malignant cancer cells. Indeed, the benefits of mTOR inhibition have been demonstrated in the clinical treatment of breast cancer (Baselga et al., 2012; Basho et al., 2018), indicating the centrality of this anabolic master switch for the both the prevention and combatting of cancer. As such, the ability of myokines released during muscular contraction to reduce mTOR activity, which has been demonstrated in other cancer types (Liu et al., 2018), presents an exciting potential mechanism by which the muscular activity inherent to physical activity may serve to negatively regulate breast cancer growth.

Recent investigations into the role and function of mTOR have yielded numerous pertinent findings, including the identification of a potent endogenous inhibitor of mTOR, the DEP Domain Containing mTOR Interacting Protein or DEPTOR (Deaver et al., 2020). DEPTOR binds to the PDZ domain of the mTOR kinase, preventing mTOR from carrying out its actions through downstream effectors. The relationship between mTOR and DEPTOR is complex; while DEPTOR potently suppresses mTOR activity (Zhao et al., 2011), the mTOR kinase is also capable of calling for the deletion of DEPTOR through activation of the β-TRCP complex (Gao et al., 2011). Studies that have investigated the role of DEPTOR in cancer indicate that DEPTOR levels are generally low across several tumor types (Caron et al., 2018), and that rescue of DEPTOR is an effective anti-cancer therapy (Xiong et al., 2018; Cuesta et al., 2019), warranting further study of this novel protein in mTOR-dependent processes.

Based on advances in the understanding of muscle-cancer cross talk, we hypothesized that pharmacologically active signaling factors released from contracting skeletal muscle would suppress anabolism in cultured MCF7 breast cancer cells by acting on the anabolic mTOR axis. We further hypothesized that this effect would be mediated by increased intracellular MCF7 DEPTOR levels. In order to test this hypothesis, we surgically isolated and perfused the hindlimb of a Wistar rat, then used electrical stimulation to produce muscular contraction of the hindlimb and collected the myokine-rich perfusate released from the hindlimb during muscle contraction. We found that this perfusate potently suppresses cancer growth in both *in vitro* and *in vivo* models of breast cancer, with perfusate acquired from untrained or moderately aerobically trained animals exhibiting similar benefits. Further, perfusate treatment profoundly suppressed both protein synthesis and anabolic signaling through the mTOR pathway, effects that were concomitant with increased DEPTOR expression. Taken together, we believe that our results conclusively demonstrate that signaling factors released from contracting skeletal muscle reduce growth and anabolism in MCF7 breast cancer cells, a process that is mediated by a suppression of mTOR activity due, at least in part, to increased levels of DEPTOR.

## Methods

### Animals and hind limb perfusion

**FIGURE 1:**
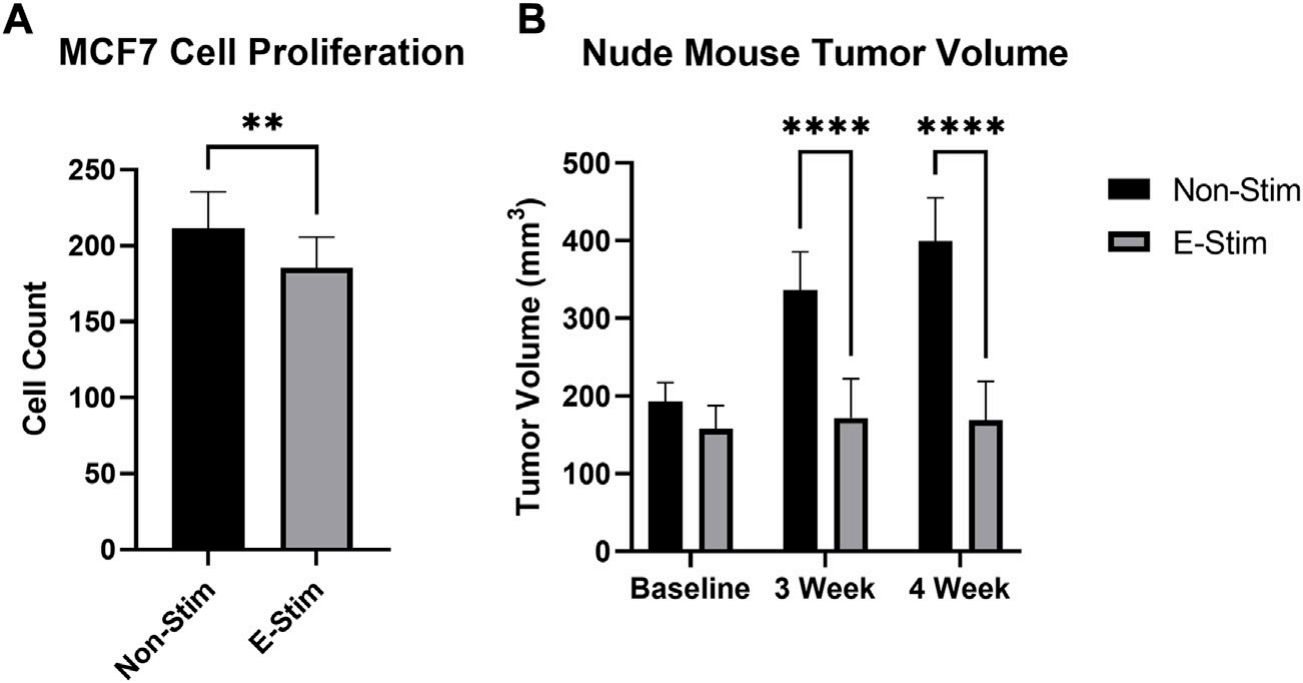
E-Stim perfusate suppresses tumor growth. Treatment with E-Stim perfusate resulted in reductions in both cell counts *in vitro* **(A)** and tumor volume *in vivo* **(B)** when compared to the Non-Stim condition. ** denotes *p* < 0.01, **** denotes *p* < 0.0001.

Female Wistar rats 8–12 weeks of age were purchased from Charles River Laboratories (Wilmington, MA). All procedures were approved by the Institutional Animal Care and Use Committee at Texas A&M University. Animals were housed two-rats per cage under standard 12 h photoperiod, provided with normal lab diet with water *ad libitum.* Following a 2 day acclimation period after arrival, animals underwent a non-survival hemicorpus hind limb perfusion preparation (HHLP) (Figure 1) as described previously (Ruderman et al., 1971). Briefly, midline to caudal end of the animal was surgically prepared so both hind limb limbs could be perfused with an oxygenated Krebs-Heinseliet buffer during electrically stimulated muscle contraction. For studies involving the role of bovine serum albumin in mediating the anti-cancer effect of exercise, albumin in the Krebs buffer was replaced with an identical amount of dextran. Electrical stimulation was administered using a stimulator (Grass Instruments, West Warwick, RI) and a force transducer (Warner Instruments, Harvard Bioscience Inc., Holliston, MA) at a surgically exposed sciatic nerve on a single hind limb of the animal. Perfusate medium was maintained at 31.7**°**C using a bipolar temperature controller (Model #CL-100, Warner Instruments, Harvard Bioscience Inc., Holliston, MA) and administered at a flow rate of 12 ml/min, by peristaltic pump (MPL 8-Channel) (Watson Marlow, Marlo, United Kingdom). One advantage of the perfusion model is the removal of the musculature from humoral input from other organ systems, including those involved with metabolism or inflammation, allowing for isolation of compounds derived specifically from the skeletal muscle.

During perfusion, medium was collected on ice in 50 ml sterile conical tubes (Corning Inc., Corning, NY). Before (Non-Stim) and during electrical stimulation (E-Stim). Following collection, samples was centrifuged at 2,500 rpm at 4**°**C to remove any remaining red blood cells, with the supernatant stored at −80**°**C until analysis.

### Exercise protocol

Female Wistar rats (*n* = 7) were exercise trained for 5 weeks. A moderate-intensity exercise was chosen for this study as it has consistently shown to be beneficial for improving and maintaining physical health (Haskell et al., 2007). The conditioning protocol utilized a motorized rodent treadmill, designed to elicit a predicted moderate intensity of 50%–75% VO_2_max, based on previous methods (Pattengale and Holloszy, 1967; Hickson et al., 1976; Burniston, 2008).

After familiarization to the treadmill and belt movement, rats were randomly assigned to the Exercise (EX) group or were assigned as age and weight matched controls (Non-EX). Forty-8 hours after the last familiarization period, rats in the EX group completed a 5-week exercise program consisting of 5 × 60min/day sessions per week. A 5–10 min warm-up and a 5 min cool-down period was included in the 60-min exercise session. The treadmill was initially fixed at 0% grade and raised at week 4%–15%; speed was gradually increased during the first 3 weeks to an average top speed of 20.5 m/min and was adjusted accordingly after the increased incline. At the conclusion of the 5-week exercise program, EX rats were perfused within 70–80 h after the last exercise bout using the rat HHLP perfusion preparation as previously described.

### Cell culture

Human MCF7 cells were obtained from American Type Culture Collection (ATCC, Manassas, VA). Cells were cultured in Dulbecco’s Modification of Eagle Medium (DMEM) (Corning, Mediatech INC., Manassas, VA), supplemented with 10% fetal bovine serum (FBS) (VWR International, Randor, PA) and 1% (v/v) Penicillin/Streptomycin (BioVision, Milpitas, CA). Cells were maintained GM at 37°C in a humidified atmosphere containing 5% CO2 until 60% confluent.

### Cell counting experiments

To determine the effects of muscle contraction *via* electrical stimulation, breast cancer cells were treated with perfusates collected from quiescent (Non-Stim) and contracting (E-Stim) skeletal muscle from the same animal (*n* = 3) *via* the HHLP preparation. Based on preliminary studies, we elected to treat cultured cells with prepared DMEM as described above, supplemented with 10% perfusate collected from either Non-Stim or E-Stim muscle. For cell counting experiments, cells were seeded in 6-well plates, with each experimental group comprising three wells. Cells were treated with Non-Stim or E-Stim perfusate for 4 days and harvested on day five. To evaluate cell proliferation, viable cells were counted using an electronic counter (Z1 Series, Beckman Coulter, Inc.), with cell counts taken in triplicate from each well.

### Western immunoblotting

In a separate set of experiments, groups of cells allocated for Western blot analysis were grown in 10 cm dishes until 60% confluent, then incubated in media containing 10% Non-Stim or E-Stim perfusate gathered in the manner described above from female Wistar rats (*n* = 4), for 24 h. All cells allocated for protein synthesis analysis were additionally treated with heavy water (^2^H_2_O) to yield a 4% final volume in order to facilitate protein fractional synthesis rate experiments (described below). After this period, an aliquot of cell media was taken, then these cells were rinsed with ice-cold phosphate buffered saline (PBS, VWR International, Randor, PA), collected using cell scrapers (VWR International, Randor, PA), and transferred to microcentrifuge tubes. These tubes were then centrifuged at 130 g for 8 min, following which PBS was aspirated and cells were flash frozen in liquid nitrogen.

For Western immunoblot experiments, cells were lysed in ice cold Norris buffer (first described in Nilsson et al., 2013). To assess the expression of proteins and their phosphorylation status, equal quantities of total protein obtained from whole cell lysates were analyzed as described previously (Fluckey et al., 2000; Nilsson et al., 2010, 2013). Proteins were resolved using SDS-PAGE, and transferred to a nitrocellulose membrane. Assessment of specific protein content was accomplished with the following antibodies: 4 E-BP1 (Cell Signaling Technology, Danvers, MA, United States #9644), Phospho-4E-BP1^Thr37/46^ (Cell Signaling #2855), P70S6K1 (Cell signaling #2708), Phospho-p70S6K1^Thr389^ (Cell Signaling #9234), and DEPTOR (Millipore Signa, Burlington, MA, United States, #ABS222). Membranes were imaged using a FlouroCHem SP imaging system (Alpha Innotech, San Leandro, CA, United States) and optical density of protein bands was determined using the Alphaease FC software (Alpha Innotech). All bands were normalized to Ponceau S staining (Romero-Calvo et al., 2010) and expressed as arbitrary units.

### Deuterium method

The validity of a deuterium approach has been demonstrated by its ability to accurately measure muscle protein synthesis (MPS) in free-living subjects over longer periods of time in order to better replicate physiological relevant conditions. The method used to assess fractional synthesis rates (FSR) in MCF7 cells was modified from the gas chromatography-mass spectroscopy (Agilent 7890 GC/5975 VL MSD, Agilent Technologies, Santa Clara, CA) method previously described (Nilsson et al., 2010).

### ^2^H_2_0 enrichment of water-soluble proteins/cell media

Briefly, cell media samples collected at harvest following 24-h of isotopic exchange were thawed on ice for 15 min. Alongside calibration standards (0–5% ^2^H_2_0, prepared by mixing naturally labeled water with 99.9% ^2^H_2_0), 20ul of cell media was aliquoted into 2 ml microcentrifuge tubes and incubated for 24-h at room temperature with 2 ul of 10 N NaOH and 4 ul of a 5% (vol/vol) solution of acetone:acetonitrile. Procedural steps from this point on were consistent as previously described (Nilsson et al., 2010). All plasma samples were measured twice with separate preparations, and an average value of the two runs were used for calculations.

### [^2^H] alanine enrichment in MCF7 cells

Cell samples were homogenized in ice-cold 10% TCA (Cl_3_CCOOH). Samples were vortexed for ∼10s before centrifugation at 3,000 rpm for 10 min s to remove unbound amino acids. Subsequently, supernatant was decanted, and the remaining cell pellet placed on ice. TCA treatment, centrifugation and decanting were repeated for a total of three spins before proceeding in accordance with previously described details procedures. Fractional synthesis rates (FSR) of mixed proteins were calculated using the equation: $E_{A}\times {\left[E_{CM}\times 3.7\times t\left(h\right)\right]}^{-1}\times 100$ *,* where E_A_ represents amount of protein-bound [^2^H]alanine (mole% excess), E_CM_ is the quantity of ^2^H_2_O in cell media (mole% excess), and 3.7 represents the exchange of ^2^H between cell media and alanine (e.g., 3.7 of 4 carbon-bound hydrogen of alanine exchange with water).

### *In Vivo* experiments

In this final set of experiments, a separate group of female Wistar rats had their hind legs perfused with oxygenated Krebs Henseleit buffer and leg muscles stimulated by electrodes placed at both ends of the limb attached to a force transducer. Contralateral muscles (SHAM) were treated identically but were not stimulated. Following stimulation, the perfusates from leg muscles were dialyzed, lyophilized and resuspended in sterile water. Based on the *in vitro* results, extracts were injected subcutaneously into female nude mice transplanted with breast tumor cells. Nu/Nu female mice age were obtained from Taconic and Charles River and kept in isolator cages. The mice receiving MCF-7 tumor cell implants were aseptically implanted in the back of the neck with slow-release Estradiol pellets (0.72 mg, 90-day pellets, Innovative Research). Tumor cells (5 × 10^6^ cells/implant) were injected into the right flank of the mice. Tumor cells were allowed to grow 1 month until average tumor size was 175 mm^3^. The animals were stratified and randomized into groups so that initial tumor size did not vary between the groups. Animals were injected daily for 4 weeks with 10 mg of perfusate resuspended in sterile water. Tumor growth was monitored and measured twice a week using a digital caliper (VWR) by investigator blinded to group assignment. Animals were then euthanized, and the tumors were removed measured and weighed.

### Statistical analysis

The effect of E-Stim *versus* Non-Stim perfusate in measures of cell proliferation, apoptosis, and protein fractional synthesis rate was assessed by use of a one-tailed *t*-test set at α > 0.05. Differences in signaling through the mTOR cascade between Non-Stim and E-Stim perfusates were analyzed by one-tailed *t*-test set at α > 0.05. A two-way ANOVA (treatment x time), with an α > 0.05 and Tukey’s post hoc test, was used for experiments involving xenografted tumor growth. All analysis was performed using GraphPad Prism, San Diego, CA United States. Figures are presented as mean ± standard error of the mean.

## Results

### Soluble factors released from contracting skeletal muscle slow growth and induce apoptosis in MCF7 breast cancer cells both *in vivo* and *in vitro*

Exposure to perfusate conditioned by a contracting rodent hindlimb (ES) led to a 12.26% reduction (Figure 1, 158.6586 vs. 139.2, *p* < 0.05) in the cell count of cultured MCF7 cells (*p* < 0.05) vs. groups treated with perfusate conditioned by a quiescent hindlimb (Figure 1). These results were replicated *in vivo,* with MCF7 tumor xenografted mice displaying reductions in tumor volume at the endpoint of the study (Figure 1). There were no differences between tumor sizes at baseline (193.2 mm^3^ vs. 157.9 mm^3^, *p* > 0.05). After 3 weeks, MCF7 xenografted mice receiving E-Stim treatment had a significant reduction in tumor size compared to Non-Stim treated mice (-48.9%, 336.3 mm^3^ vs. 171.8 mm^3^, *p* < 0.05), an effect that persisted into the fourth week (-57.7%, 399.5 mm^3^ vs. 169.1 mm^3^, *p* < 0.05). Based on these *in vitro* and *in vivo* results, we conclude that contracting skeletal muscle releases a pharmacologically active factor which exerts a powerful anti-cancer effect in MCF7 breast cancer cells, to such a degree that *in vivo* tumors treated with E-Stim perfusate displayed virtually no growth.

### Anti-cancer cross-talk between skeletal muscle and MCF7 cells is mechanistically mediated by reduced anabolism and mTOR signaling

**FIGURE 2:**
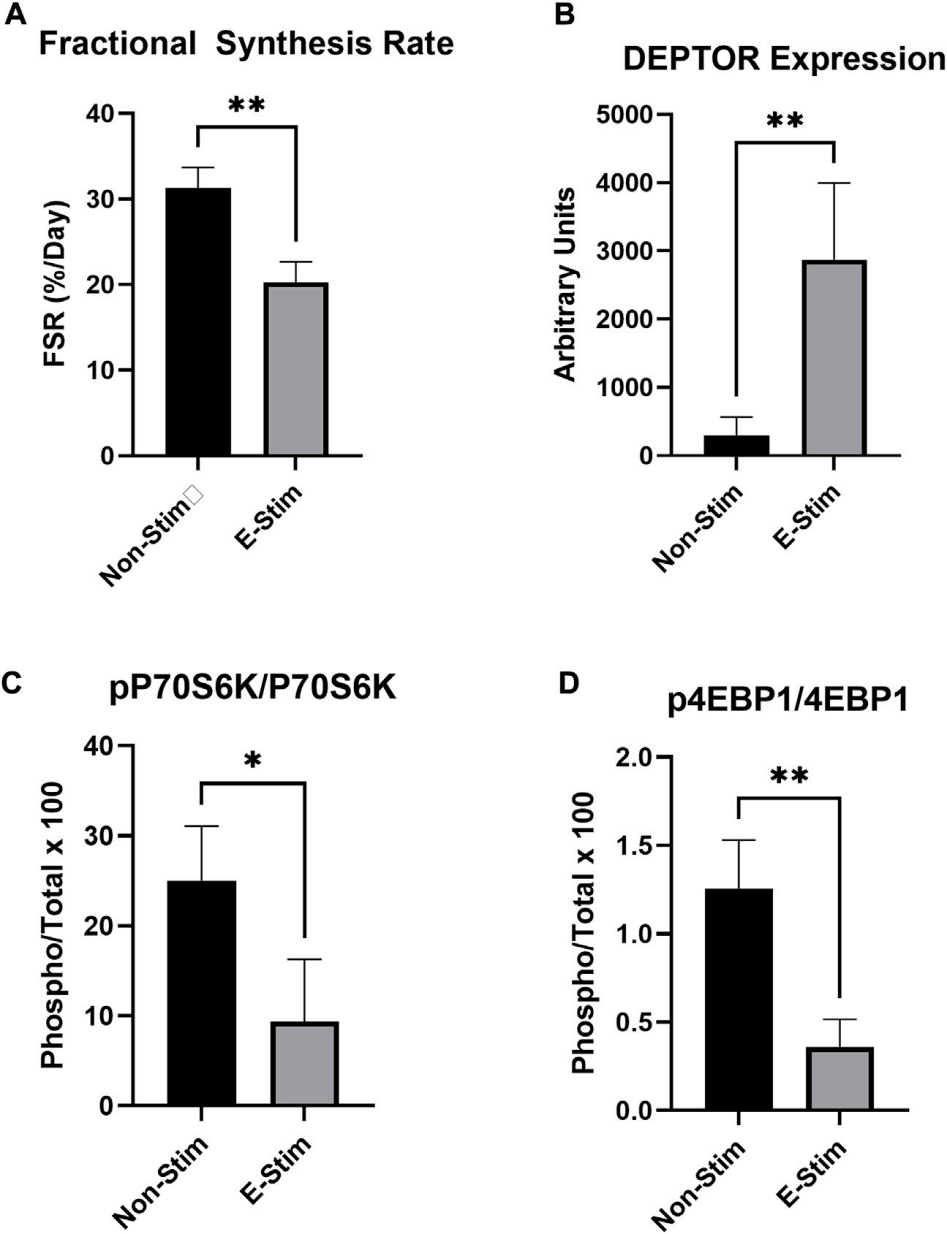
E-Stim perfusate suppresses anabolic signaling in MCF7 breast cancer cells. Cellular protein fractional synthesis rate was reduced by E-Stim perfusate treatment **(A)**. E-Stim perfusate also lead to an accumulation of the endogenous mTOR inhibitor DEPTOR **(B)**, concomitant with reduced signaling through the downstream targets of the anabolic mTOR kinase **(C,D)**. * denotes *p* < 0.05, ** denotes *p* < 0.01.

Stable-label isotope tracer experiments utilizing deuterium oxide demonstrate that 24-h rates of protein synthesis were reduced by 35% in MCF7 cells receiving E-Stim perfusate treatment (31.33%/day vs. 20.30%/day, *p* < 0.05) (Figure 2). These reductions of fractional synthesis rate were accompanied by reductions in the ratio of phosphorylated to total P70S6K (−71.30%, 1.25 vs. 0.36, *p* < 0.05) and 4EBP1 (−62.57%, 20.05 vs. 7.51, *p* < 0.05), two downstream targets of the anabolic mTOR kinase. Underlying this observation, expression of the endogenous mTOR inhibitor, DEPTOR, was increased by nearly 900% (296 vs. 2874 A.U. *p* < 0.05) in E-Stim perfusate treated cells over Non-Stim treated groups, suggesting that E-Stim perfusate slows anabolism in MCF7 breast cancer *via* action on the mTOR signaling cascade.

### The effect of myokine treatment is not altered by habitual exercise

**FIGURE 3:**
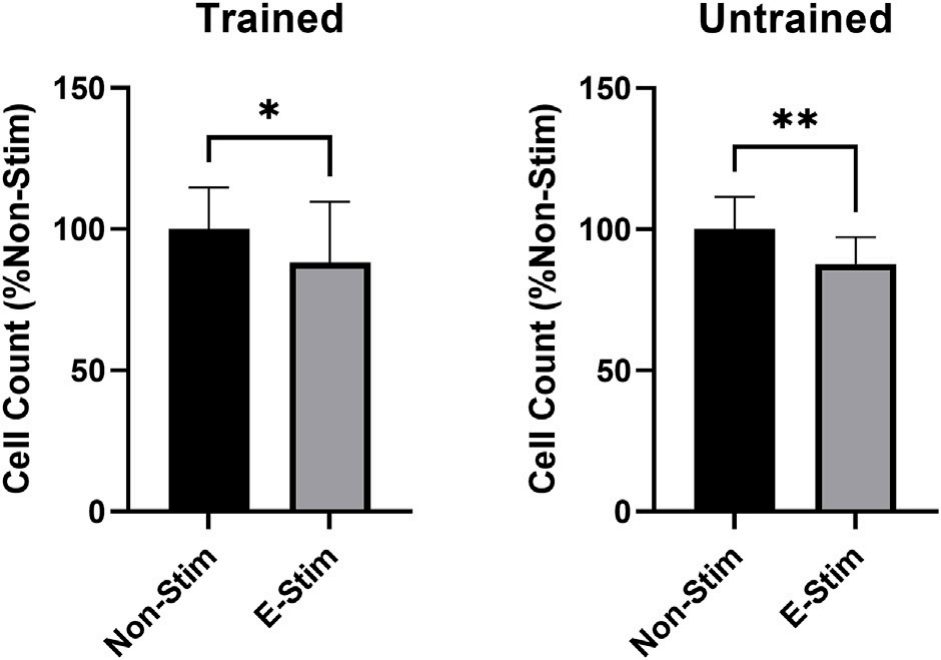
Habitual exercise does not alter the effect of E-Stim perfusate on breast cancer cell growth. E-Stim perfusates gathered from both trained and untrained animals led to similar reductions in MCF7 cell count. * denotes *p* < 0.05, ** denotes *p* < 0.01.

In order to investigate the role of prior exercise training on the effect contraction-derived myokines on MCF7 cancer cell growth, perfusates were collected from rats exposed to 5 weeks of aerobic exercise training and age/weight matched controls. Conditioned perfusate was then collected from these animals, and subsequent analysis of proliferation performed, in a manner identical to that used for untrained animals. When comparing the effect of E-Stim and Non-Stim perfusate from trained animals with that of the untrained, we found that trained E-Stim perfusate resulted in a near replication of the prior result–an 11.77% reduction (Figure 3) in cell count in the trained animal experiments (57.64 vs. 50.86, *p* < 0.05). These data indicate that prior exercise training is not important for the anti-proliferative benefits of contracting muscle on breast cancer, and that the release of soluble factors mediating the mechanistic anti-cancer benefit of exercise is an inherent property of muscular contraction, independent of exercise training status.

### Albumin is required in the perfusate for the effects of exercise on breast cancer

**FIGURE 4:**
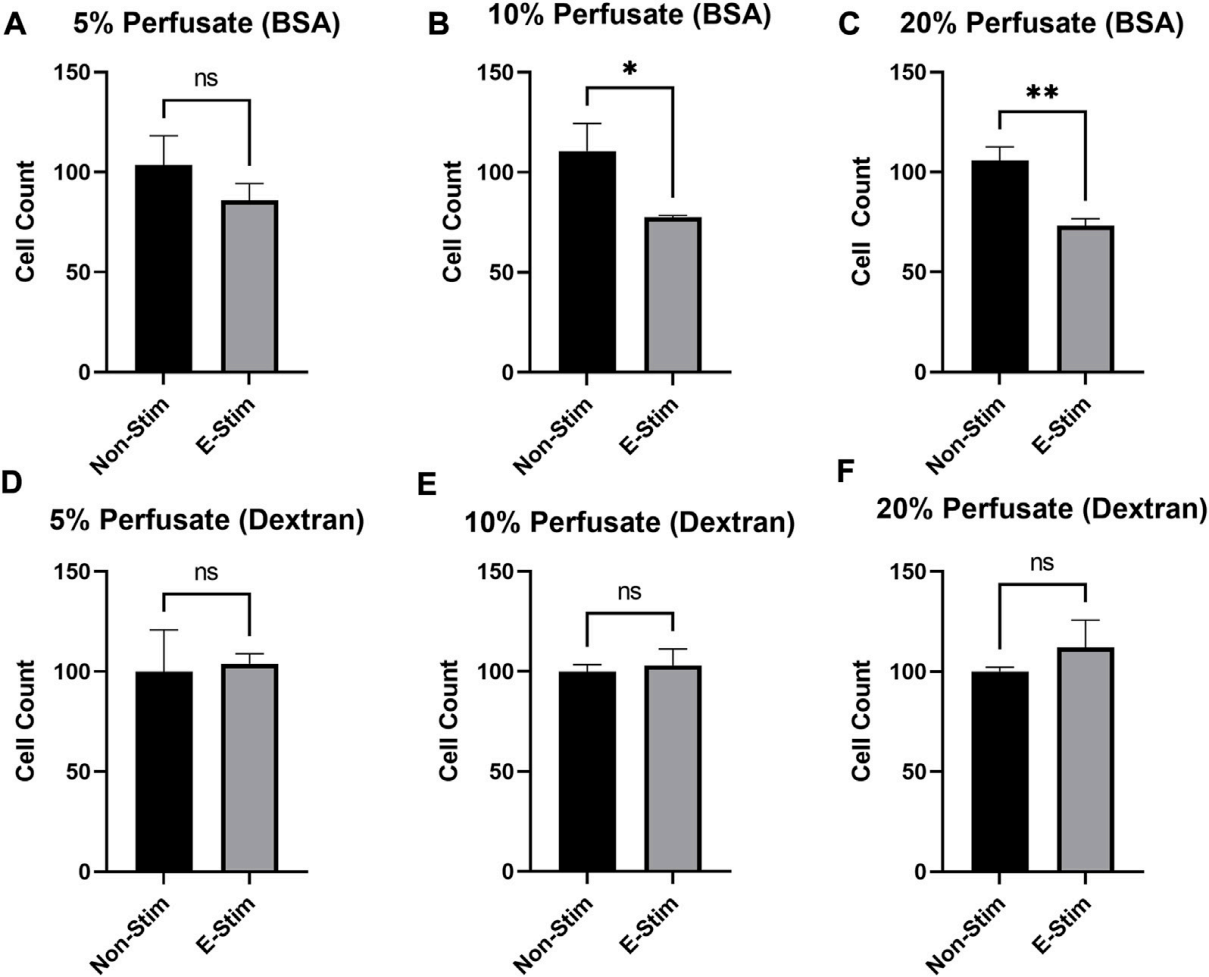
Albumin is required to mediate the chemotherapeutic effects of E-Stim perfusate. In perfusates where albumin **(A−C)** was substituted with dextran **(D−F)**, no effect of E-Stim perfusate treatment on MCF7 cell count was observed. The purpose of this experiment was to determine if albumin in the perfusate had a role in delivering myokines through the vasculature. * denotes *p* < 0.05, ** denotes *p* < 0.01.

In order to examine the effect that circulating proteins play in mediating the effects of muscle-derived myokines on breast cancer, perfusates were prepared either with bovine serum albumin (BSA) or dextran, with the parameters of the contraction and collection remaining identical. We found (Figure 4) that there was a dose-dependent relationship between perfusate (prepared with BCA) concentration and inhibition of cell growth, such that a minimum of 10% supplementation with exercise–conditioned perfusate was required to induce a 33.11% reduction in MCF7 cell count in our early preliminary experiments (110.6 vs. 77.56, *p* < 0.05). However, no amount (5%, 10%, or 20%) of media supplemented with dextran-prepared perfusate led to a reduction in MCF7 cell proliferation (*p* > 0.05). Thus, we conclude that the presence of albumin is in some way required to mediate the observed benefit of contraction-derived myokines on cancer cell growth.

## Discussion

Breast cancer is a major cause of morbidity and mortality for over 2.8 million women in the United States, presenting a pressing need to identify routes of both prevention and treatment for these deadly tumors. It has long been appreciated that regular physical exercise, as part of an overall healthy lifestyle, is effective in combatting many chronic diseases, including the development and recurrence of cancer (Bernstein et al., 1994; Carpenter et al., 1999; Cormie et al., 2017; Hojman et al., 2018). While exercise certainly has systemic benefits that are advantageous for overall health, results from the present study support a growing body of literature (Hojman et al., 2011, 2018; Aoi et al., 2013; Gannon et al., 2015; Dethlefsen et al., 2016; Ruiz-Casado et al., 2017) which suggest that contracting skeletal muscle possesses the ability to regulate the metabolism of cancer cells through direct mechanistic cross-talk. Our findings indicate that some pharmacologically active factor or factors are secreted from contracting skeletal muscle, and that these compounds have demonstrable effects on the growth and metabolism of cultured breast cancer cells. By acting on the well-documented anabolic mTOR axis, myokines released during muscle contraction slow proliferation, induce apoptosis, and suppress protein synthesis in MCF7 cancer cells, both *in vitro* and *in vivo,* to the extent that exercise-conditioned perfusate treatment in mice with xenografted tumors completely abrogates tumor growth at three- and 4-weeks post-implantation. These results support the hypothesis that skeletal muscle is capable of altering the behavior of extra-muscular tissues, and that the act of physical exercise can, *per se,* provide demonstrable benefits in combating malignancies of the breast.

The mTOR signaling pathway is central to multiple cell cycle and survival mechanisms, and serves to regulate cell growth, cellular proliferation and survival, all of which are vital for the function of a healthy cell (Deaver et al., 2020). While mTOR is critical to the normal function of the cell, dysregulation of mTOR is common in tumors of the breast (Paplomata and O’Regan, 2014; Hare and Harvey, 2017), where a constitutively active mTOR plays a role in several aspects of tumor pathology, including the rapid proliferation and resistance to apoptosis that are among the hallmarks of cancer (Cargnello et al., 2015). mTOR ultimately acts on two downstream effectors, P70S6K and 4EBP1 to promote cap-dependent mRNA translation, with the subsequently manufactured proteins playing a role in a number of proliferative and anabolic processes. Thus, the finding that exercise-conditioned perfusate treatment resulted in significantly diminished mTOR activity, as measured by Western immunoblot of its substrates, P70S6K and 4EBP1, and marked suppression of protein synthesis, is especially exciting, and provides evidence of a novel means by which signaling released from skeletal muscle may act on this canonical signaling pathway to control anabolism in a cancerous cell.

A potent inhibitor of the mTOR enzymatic complex is the DEP domain-containing mTOR-interacting protein (DEPTOR) (Caron et al., 2018). The dynamic between mTOR and DEPTOR is a matter of ongoing research, but what is clear is that activation of mTOR leads to phosphorylation and subsequent degradation of DEPTOR, allowing the full activation of the former (Duan et al., 2011; Gao et al., 2011). The DEPTOR accumulation and a subsequent reduction in mTOR activity as a result of perfusate treatment is therefore of great interest, with ongoing experiments seeking to further investigate this mechanism. It is well-documented that DEPTOR expression is low in most cancers (Wang et al., 2012; Li et al., 2014; Chen et al., 2020), including those of the breast (Bi et al., 2021). However, to our knowledge, this study is the first to demonstrate that exposure of epithelial breast cancer cells to medium collected from contracting skeletal muscles results in a rescue of DEPTOR within the cancer cell, blunting mTOR signaling and with consequent reductions in protein synthesis. Taken together, these results demonstrate, for the first time, that factors released from skeletal muscle suppress anabolism in breast cancer *via* action on the canonical mTOR pathway.

Although the present paper cannot determine what factors led to the reduction of mTOR activity and cancer cell proliferation, it is possible this effect could be due to factors in the perfusate that can modify transcriptional/translational behavior in the cell, as evidenced by the rescued expression of DEPTOR. Recent evidence suggests that the transfer of mircroRNA (miRNA), small strands of nucleotides capable of altering cellular behavior and metabolism through the process of RNA interference (Hannon, 2002; Chen et al., 2012; Reddy, 2015), between cells is a means for intercellular communication (Chen et al., 2012; Hamam et al., 2017). While we are currently in the process of characterizing the myokines released during muscular contraction, the finding that albumin in the perfusate at a certain threshold is required for the chemotherapeutic effect of E-Stim on breast cancer provides some tantalizing clues as to the nature of this phenomenon. Although much work in identifying the means by which these compounds are released from parent cells, transported through the body, and taken up by target cells remains to be done, some investigations have demonstrated that the miRNA release and subsequent uptake may be mediated by nucleoprotein complexes (Arroyo et al., 2011; Hamam et al., 2017). There is growing evidence for this mechanism, and the finding that albumin protein is necessary to mediate the anti-cancer effects of the exercise condition perfusate in our experiments is especially interesting, given recent studies showing that serum albumin displays discrete binding regions for miRNA (Alinovskaya et al., 2018).

Intriguingly, the observed benefits of muscle contraction on cancer growth were similar when examining the effect of perfusates acquired from the contracting hindlimb of both sedentary and aerobic exercise-trained animals, strongly suggesting that the factors responsible for mediating the mechanistic anti-cancer effect of exercise is inherently resident in muscle, and that its manufacture and release is tied to muscular contraction *per se*, independent of physical conditioning. This carries an interesting clinical application: that any individual may benefit from the repeated muscle contractions that constitute exercise, regardless of prior physical conditioning, and highlights the need for physical activity to stimulate the secretion of factors that combat cancer. While the protocol used for inducing exercise adaptations represents a fairly moderate aerobic training program, it has been demonstrated to lead to appreciable training adaptations in animal models. More investigation on the role of training in the anti-cancer benefits of exercise is clearly required; but our results strongly suggest that the mechanistic factors mediating these benefits are normally resident in muscle, and that their manufacture and subsequent release are completely independent of physical conditioning. Perhaps our most exciting finding is that any individual may benefit from repeated muscle contractions, regardless of prior physical conditioning, highlighting the need for physical activity to stimulate the secretion of factors that combat cancer.

Consistent with epidemiological and physiological studies demonstrating an attenuated risk and progression of breast cancer with exercise (Lee, 2003; Westerlind et al., 2003; Hayes et al., 2018), our results support the concept that the very act of locomotion (i.e., contracting skeletal muscle) has a direct therapeutic influence on breast cancer. Our work expands on that of others by using a hind limb perfusion preparation, where we can effectively remove other humoral factors, such as hormones or cytokines from other organs, that may also impact growing cancers, and administer that perfusate directly to the cancer cells. We believe the model of exercise used here allowed us to successfully isolate myokines that are reflective of what the muscle is capable of releasing *in vivo*, in order to allow us to investigate the specific mechanistic effects of contraction-derived myokines on cancer cell metabolism. However, while the acute exercise model of electrical stimulation *via* the sciatic nerve may be more physiologically correct than non-voluntary stimulation by other means, this model is still not completely physiological. True neural activation during *in vivo* exercise activates the motor units in a proportional fashion to match the forces required to meet the demand of the activity. Our model simultaneously activated all the motor units of the lower limb musculature, which is not typical of most exercise in humans. This approach capitalizes on previous findings that this stimulation paradigm maximizes glucose uptake in all muscles of the leg, with the rationale that contraction-mediated glucose transporter translocation (specifically, GLUT-4) and fusion with the surface membranes requires exocytosis of intracellular vesicles (Richter and Hargreaves, 2013). Along that line of thinking, we feel that the present study may warrant experiments involving resistance exercise programs, where simultaneous activation of larger muscle groups may provide more of a stimulatory effect. Thus, we contend that while a fully *in vivo* assessment of the interaction of myokines and breast cancer exercise is warranted, our findings strongly point to the fact that muscle contractions release substances that negatively impact cancer cells.

In summary, we demonstrate that the protective benefits of exercise against breast cancer can be attributed, at least in part, to myokines released by contracting skeletal muscle, independent of any hormonal or whole-body effects of regular physical activity. These myokines suppressed cancer growth independent of whether they were derived from the muscles of a sedentary or active animal, indicating that the release of anti-cancer myokines is an inherent property of muscular contraction. These compounds act on the anabolic mTOR axis of the cancer cell, suppressing protein synthesis and rescuing the endogenous inhibitor DEPTOR. We recognize that there are numerous forms of breast cancers in clinical populations and understand the importance of conducting similar studies across that spectrum to further validate the findings in this study. Ongoing experiments in our laboratory are devoted to pursuing the identity of which specific molecules responsible for mediating this effect, the exact nature of their release from the muscle, and how they are taken up by cancerous cells of the breast. Taken together, we believe that further investigation of the relationship between skeletal muscle, myokines, and breast cancer will provide exciting novel outcomes, and pave the way for clinical advances in the understanding and treatment of malignant tumors.

## Data Availability

The raw data supporting the conclusion of this article will be made available by the authors, without undue reservation.

## References

[B1] Alinovskaya L. I. Sedykh S. E. Ivanisenko N. V. Soboleva S. E. Nevinsky G. A. (2018). How human serum albumin recognizes DNA and RNA. Biol. Chem. 399, 347–360. 10.1515/hsz-2017-0243 29252186

[B2] Aoi W. Naito Y. Takagi T. Tanimura Y. Takanami Y. Kawai Y. (2013). A novel myokine, secreted protein acidic and rich in cysteine (SPARC), suppresses colon tumorigenesis via regular exercise. Gut 62, 882–889. 10.1136/gutjnl-2011-300776 22851666

[B3] Arroyo J. D. Chevillet J. R. Kroh E. M. Ruf I. K. Pritchard C. C. Gibson D. F. (2011). Argonaute2 complexes carry a population of circulating microRNAs independent of vesicles in human plasma. Proc. Natl. Acad. Sci. U. S. A. 108, 5003–5008. 10.1073/pnas.1019055108 21383194 PMC3064324

[B4] Baselga J. Campone M. Piccart M. Burris H. A. Rugo H. S. Sahmoud T. (2012). Everolimus in postmenopausal hormone-receptor–positive advanced breast cancer. N. Engl. J. Med. 366, 520–529. 10.1056/NEJMoa1109653 22149876 PMC5705195

[B5] Basho R. K. Yam C. Gilcrease M. Murthy R. K. Helgason T. Karp D. D. (2018). Comparative effectiveness of an mTOR‐based systemic therapy regimen in advanced, metaplastic and nonmetaplastic triple‐negative breast cancer. Oncologist 23, 1300–1309. 10.1634/theoncologist.2017-0498 30139837 PMC6291334

[B6] Bernstein L. Henderson B. E. Hanisch R. Sullivan-Halley J. Ross R. K. (1994). Physical exercise and reduced risk of breast cancer in young women. J. Natl. Cancer Inst. 86, 1403–1408. 10.1093/jnci/86.18.1403 8072034

[B7] Bernstein L. Patel A. V. Ursin G. Sullivan-Halley J. Press M. F. Deapen D. (2005). Lifetime recreational exercise activity and breast cancer risk among black women and white women. J. Natl. Cancer Inst. 97, 1671–1679. 10.1093/jnci/dji374 16288120

[B8] Bi Y. Gong L. Liu P. Xiong X. Zhao Y. (2021). Nuclear ErbB2 represses DEPTOR transcription to inhibit autophagy in breast cancer cells. Cell Death Dis. 12, 397–406. 10.1038/s41419-021-03686-9 33854045 PMC8047043

[B9] Brown J. C. Kontos D. Schnall M. D. Wu S. Schmitz K. H. (2016). The dose-response effects of aerobic exercise on body composition and breast tissue among women at high risk for breast cancer: A randomized trial. Cancer Prev. Res. 9, 581–588. 10.1158/1940-6207.CAPR-15-0408 PMC5350624 27099272

[B10] Burniston J. G. (2008). Changes in the rat skeletal muscle proteome induced by moderate-intensity endurance exercise. Biochim. Biophys. Acta 1784, 1077–1086. 10.1016/j.bbapap.2008.04.007 18482594

[B11] Cargnello M. Tcherkezian J. Roux P. P. (2015). The expanding role of mTOR in cancer cell growth and proliferation. Mutagenesis 30, 169–176. 10.1093/mutage/geu045 25688110 PMC5943824

[B12] Caron A. Briscoe D. M. Richard D. Laplante M. (2018). DEPTOR at the nexus of cancer, metabolism, and immunity. Physiol. Rev. 98, 1765–1803. 10.1152/physrev.00064.2017 29897294 PMC6335100

[B13] Carpenter C. L. Ross R. K. Paganini-Hill A. Bernstein L. (1999). Lifetime exercise activity and breast cancer risk among post-menopausal women. Br. J. Cancer 80, 1852–1858. 10.1038/sj.bjc.6690610 10468309 PMC2374273

[B15] Chang J. S. Kim T. H. Kong I. D. (2020). Exercise intervention lowers aberrant serum WISP-1 levels with insulin resistance in breast cancer survivors: A randomized controlled trial. Sci. Rep. 10, 10898. 10.1038/s41598-020-67794-w 32616883 PMC7331642

[B16] Chen W. Wang L. You W. Shan T. (2021). Myokines mediate the cross talk between skeletal muscle and other organs. J. Cell. Physiol. 236, 2393–2412. 10.1002/jcp.30033 32885426

[B17] Chen X. Liang H. Zhang J. Zen K. Zhang C.-Y. (2012). Secreted microRNAs: A new form of intercellular communication. Trends Cell Biol. 22, 125–132. 10.1016/j.tcb.2011.12.001 22260888

[B18] Chen X. Lu W. Zheng W. Gu K. Matthews C. E. Chen Z. (2011). Exercise after diagnosis of breast cancer in association with survival. Cancer Prev. Res. 4, 1409–1418. 10.1158/1940-6207.CAPR-10-0355 PMC3169008 21795422

[B19] Chen X. Xiong X. Cui D. Yang F. Wei D. Li H. (2020). DEPTOR is an *in vivo* tumor suppressor that inhibits prostate tumorigenesis via the inactivation of mTORC1/2 signals. Oncogene 39, 1557–1571. 10.1038/s41388-019-1085-y 31685947 PMC7018663

[B20] Colaianni G. Cuscito C. Mongelli T. Pignataro P. Buccoliero C. Liu P. (2015). The myokine irisin increases cortical bone mass. Proc. Natl. Acad. Sci. U. S. A. 112, 12157–12162. 10.1073/pnas.1516622112 26374841 PMC4593131

[B21] Cormie P. Zopf E. M. Zhang X. Schmitz K. H. (2017). The impact of exercise on cancer mortality, recurrence, and treatment-related adverse effects. Epidemiol. Rev. 39, 71–92. 10.1093/epirev/mxx007 28453622

[B22] Cuesta R. Gritsenko M. A. Petyuk V. A. Shukla A. K. Tsai C.-F. Liu T. (2019). Phosphoproteome analysis reveals estrogen-ER pathway as a modulator of mTOR activity via DEPTOR. Mol. Cell. Proteomics 18, 1607–1618. 10.1074/mcp.RA119.001506 31189691 PMC6683011

[B23] de Roon M. May A. M. McTiernan A. Scholten R. J. P. M. Peeters P. H. M. Friedenreich C. M. (2018). Effect of exercise and/or reduced calorie dietary interventions on breast cancer-related endogenous sex hormones in healthy postmenopausal women. Breast Cancer Res. 20, 81. 10.1186/s13058-018-1009-8 30071893 PMC6090977

[B24] Deaver J. W. López S. M. Ryan P. J. Nghiem P. P. Riechman S. E. Fluckey J. D. (2020). Regulation of cellular anabolism by mTOR: or how I learned to stop worrying and love translation. Sports Med. Health Sci. 2, 195–201. 10.1016/j.smhs.2020.11.003 35782997 PMC9219308

[B25] Dethlefsen C. Hansen L. S. Lillelund C. Andersen C. Gehl J. Christensen J. F. (2017). Exercise-Induced catecholamines activate the hippo tumor suppressor pathway to reduce risks of breast cancer development. Cancer Res. 77, 4894–4904. 10.1158/0008-5472.CAN-16-3125 28887324

[B26] Dethlefsen C. Lillelund C. Midtgaard J. Andersen C. Pedersen B. K. Christensen J. F. (2016). Exercise regulates breast cancer cell viability: Systemic training adaptations versus acute exercise responses. Breast Cancer Res. Treat. 159, 469–479. 10.1007/s10549-016-3970-1 27601139

[B27] Duan S. Skaar J. R. Kuchay S. Toschi A. Kanarek N. Ben-Neriah Y. (2011). mTOR generates an auto-amplification loop by triggering the βTrCP- and CK1α-dependent degradation of DEPTOR. Mol. Cell 44, 317–324. 10.1016/j.molcel.2011.09.005 22017877 PMC3212871

[B28] Febbraio M. A. Hiscock N. Sacchetti M. Fischer C. P. Pedersen B. K. (2004). Interleukin-6 is a novel factor mediating glucose homeostasis during skeletal muscle contraction. Diabetes 53, 1643–1648. 10.2337/diabetes.53.7.1643 15220185

[B29] Fluckey J. D. Pohnert S. C. Boyd S. G. Cortright R. N. Trappe T. A. Dohm G. L. (2000). Insulin stimulation of muscle protein synthesis in obese Zucker rats is not via a rapamycin-sensitive pathway. Am. J. Physiol. Endocrinol. Metab. 279, E182–E187. 10.1152/ajpendo.2000.279.1.E182 10893338

[B30] Friedenreich C. M. Woolcott C. G. McTiernan A. Ballard-Barbash R. Brant R. F. Stanczyk F. Z. (2010). Alberta physical activity and breast cancer prevention trial: Sex hormone changes in a year-long exercise intervention among postmenopausal women. J. Clin. Oncol. 28, 1458–1466. 10.1200/JCO.2009.24.9557 20159820 PMC2849767

[B31] Gannon N. P. Vaughan R. A. Garcia‐Smith R. Bisoffi M. Trujillo K. A. (2015). Effects of the exercise-inducible myokine irisin on malignant and non-malignant breast epithelial cell behavior *in vitro* . Int. J. Cancer 136, E197–E202. 10.1002/ijc.29142 25124080

[B32] Gao D. Inuzuka H. Tan M.-K. M. Fukushima H. Locasale J. W. Liu P. (2011). mTOR drives its own activation via SCFβTrCP-dependent degradation of the mTOR inhibitor DEPTOR. Mol. Cell 44, 290–303. 10.1016/j.molcel.2011.08.030 22017875 PMC3229299

[B33] Hamam R. Hamam D. Alsaleh K. A. Kassem M. Zaher W. Alfayez M. (2017). Circulating microRNAs in breast cancer: Novel diagnostic and prognostic biomarkers. Cell Death Dis. 8, e3045. 10.1038/cddis.2017.440 28880270 PMC5636984

[B34] Hannon G. J. (2002). RNA interference. Nature 418, 244–251. 10.1038/418244a 12110901

[B35] Hare S. H. Harvey A. J. (2017). mTOR function and therapeutic targeting in breast cancer. Am. J. Cancer Res. 7, 383–404. 28400999 PMC5385631

[B36] Haskell W. L. Lee I.-M. Pate R. R. Powell K. E. Blair S. N. Franklin B. A. (2007). Physical activity and public health: Updated recommendation for adults from the American College of Sports medicine and the American heart association. Med. Sci. Sports Exerc. 39, 1423–1434. 10.1249/mss.0b013e3180616b27 17762377

[B37] Hayes S. C. Steele M. L. Spence R. R. Gordon L. Battistutta D. Bashford J. (2018). Exercise following breast cancer: Exploratory survival analyses of two randomised, controlled trials. Breast Cancer Res. Treat. 167, 505–514. 10.1007/s10549-017-4541-9 29063309

[B38] Hickson R. C. Heusner W. W. Van Huss W. D. (1976). Skeletal muscle enzyme alterations after sprint and endurance training. J. Appl. Physiol. 40, 868–871. 10.1152/jappl.1976.40.6.868 931923

[B39] Hoffmann C. Weigert C. (2017). Skeletal muscle as an endocrine organ: The role of myokines in exercise adaptations. Cold Spring Harb. Perspect. Med. 7, a029793. 10.1101/cshperspect.a029793 28389517 PMC5666622

[B40] Hojman P. Dethlefsen C. Brandt C. Hansen J. Pedersen L. Pedersen B. K. (2011). Exercise-induced muscle-derived cytokines inhibit mammary cancer cell growth. Am. J. Physiol. Endocrinol. Metab. 301, E504–E510. 10.1152/ajpendo.00520.2010 21653222

[B41] Hojman P. Gehl J. Christensen J. F. Pedersen B. K. (2018). Molecular mechanisms linking exercise to cancer prevention and treatment. Cell Metab. 27, 10–21. 10.1016/j.cmet.2017.09.015 29056514

[B42] Holmes M. D. Chen W. Y. Feskanich D. Kroenke C. H. Colditz G. A. (2005). Physical activity and survival after breast cancer diagnosis. JAMA 293, 2479–2486. 10.1001/jama.293.20.2479 15914748

[B43] Iwase T. Wang X. Shrimanker T. V. Kolonin M. G. Ueno N. T. (2021). Body composition and breast cancer risk and treatment: Mechanisms and impact. Breast Cancer Res. Treat. 186, 273–283. 10.1007/s10549-020-06092-5 33475878

[B44] Lee I.-M. (2003). Physical activity and cancer prevention--data from epidemiologic studies. Med. Sci. Sports Exerc. 35, 1823–1827. 10.1249/01.MSS.0000093620.27893.23 14600545

[B45] Li H. Sun G. Y. Zhao Y. Thomas D. Greenson J. K. Zalupski M. M. (2014). DEPTOR has growth suppression activity against pancreatic cancer cells. Oncotarget 5, 12811–12819. 10.18632/oncotarget.2659 25544749 PMC4350351

[B46] Liu J. Song N. Huang Y. Chen Y. (2018). Irisin inhibits pancreatic cancer cell growth via the AMPK-mTOR pathway. Sci. Rep. 8, 15247. 10.1038/s41598-018-33229-w 30323244 PMC6189061

[B47] Meneses-Echávez J. F. Correa-Bautista J. E. González-Jiménez E. Schmidt Río-Valle J. Elkins M. R. Lobelo F. (2016). The effect of exercise training on mediators of inflammation in breast cancer survivors: A systematic review with meta-analysis. Cancer Epidemiol. Biomarkers Prev. 25, 1009–1017. 10.1158/1055-9965.EPI-15-1061 27197276

[B48] Mossmann D. Park S. Hall M. N. (2018). mTOR signalling and cellular metabolism are mutual determinants in cancer. Nat. Rev. Cancer 18, 744–757. 10.1038/s41568-018-0074-8 30425336

[B49] Murphy E. A. Davis J. M. Barrilleaux T. L. McClellan J. L. Steiner J. L. Carmichael M. D. (2011). Benefits of exercise training on breast cancer progression and inflammation in C3(1)SV40Tag mice. Cytokine 55, 274–279. 10.1016/j.cyto.2011.04.007 21600785 PMC3383660

[B50] Nilsson M. I. Dobson J. P. Greene N. P. Wiggs M. P. Shimkus K. L. Wudeck E. V. (2013). Abnormal protein turnover and anabolic resistance to exercise in sarcopenic obesity. FASEB J. 27, 3905–3916. 10.1096/fj.12-224006 23804240

[B51] Nilsson M. I. Greene N. P. Dobson J. P. Wiggs M. P. Gasier H. G. Macias B. R. (2010). Insulin resistance syndrome blunts the mitochondrial anabolic response following resistance exercise. Am. J. Physiol. Endocrinol. Metab. 299, E466–E474. 10.1152/ajpendo.00118.2010 20606077

[B52] Paplomata E. O’Regan R. (2014). The PI3K/AKT/mTOR pathway in breast cancer: Targets, trials and biomarkers. Ther. Adv. Med. Oncol. 6, 154–166. 10.1177/1758834014530023 25057302 PMC4107712

[B53] Pattengale P. Holloszy J. (1967). Augmentation of skeletal muscle myoglobin by a program of treadmill running. Am. J. Physiol. 213, 783–785. 10.1152/ajplegacy.1967.213.3.783 6036801

[B54] Pedersen B. K. Åkerström T. C. A. Nielsen A. R. Fischer C. P. (2007). Role of myokines in exercise and metabolism. J. Appl. Physiol. 103, 1093–1098. 10.1152/japplphysiol.00080.2007 17347387

[B55] Pedersen B. K. (2011). Exercise-induced myokines and their role in chronic diseases. Brain Behav. Immun. 25, 811–816. 10.1016/j.bbi.2011.02.010 21354469

[B56] Raschke S. Eckardt K. Holven K. B. Jensen J. Eckel J. (2013). Identification and validation of novel contraction-regulated myokines released from primary human skeletal muscle cells. PLOS ONE 8, e62008. 10.1371/journal.pone.0062008 23637948 PMC3634789

[B57] Reddy K. B. (2015). MicroRNA (miRNA) in cancer. Cancer Cell Int. 15, 38. 10.1186/s12935-015-0185-1 25960691 PMC4424445

[B58] Richter E. A. Hargreaves M. (2013). Exercise, GLUT4, and skeletal muscle glucose uptake. Physiol. Rev. 93, 993–1017. 10.1152/physrev.00038.2012 23899560

[B59] Romero-Calvo I. Ocón B. Martínez-Moya P. Suárez M. D. Zarzuelo A. Martínez-Augustin O. (2010). Reversible Ponceau staining as a loading control alternative to actin in Western blots. Anal. Biochem. 401, 318–320. 10.1016/j.ab.2010.02.036 20206115

[B60] Ruderman N. B. Houghton C. R. S. Hems R. (1971). Evaluation of the isolated perfused rat hindquarter for the study of muscle metabolism. Biochem. J. 124, 639–651. 10.1042/bj1240639 5135248 PMC1177234

[B61] Ruiz-Casado A. Martín-Ruiz A. Pérez L. M. Provencio M. Fiuza-Luces C. Lucia A. (2017). Exercise and the hallmarks of cancer. Trends Cancer 3, 423–441. 10.1016/j.trecan.2017.04.007 28718417

[B62] Saxton R. A. Sabatini D. M. (2017). mTOR signaling in growth, metabolism, and disease. Cell 168, 960–976. 10.1016/j.cell.2017.02.004 28283069 PMC5394987

[B63] Schwappacher R. Dieterich W. Reljic D. Pilarsky C. Mukhopadhyay D. Chang D. K. (2021). Muscle-derived cytokines reduce growth, viability and migratory activity of pancreatic cancer cells. Cancers 13, 3820. 10.3390/cancers13153820 34359731 PMC8345221

[B64] Severinsen M. C. K. Pedersen B. K. (2020). Muscle–organ crosstalk: The emerging roles of myokines. Endocr. Rev. 41, bnaa016–609. 10.1210/endrev/bnaa016 32393961 PMC7288608

[B65] Shephard R. J. (1993). Exercise in the prevention and treatment of cancer. An update. Sports Med. 15, 258–280. 10.2165/00007256-199315040-00004 8460289

[B66] Siegel R. L. Miller K. D. Jemal A. (2020). Cancer statistics, 2020. Ca. Cancer J. Clin. 70, 7–30. 10.3322/caac.21590 31912902

[B67] Thune I. Brenn T. Lund E. Gaard M. (1997). Physical activity and the risk of breast cancer. N. Engl. J. Med. 336, 1269–1275. 10.1056/NEJM199705013361801 9113929

[B68] Wang Z. Zhong J. Inuzuka H. Gao D. Shaik S. Sarkar F. H. (2012). An evolving role for DEPTOR in tumor development and progression. Neoplasia 14, 368–375. 10.1593/neo.12542 22745583 PMC3384424

[B69] Westerlind K. C. McCarty H. L. Schultheiss P. C. Story R. Reed A. H. Baier M. L. (2003). Moderate exercise training slows mammary tumour growth in adolescent rats. Eur. J. Cancer Prev. 12, 281–287. 10.1097/00008469-200308000-00007 12883380

[B70] Westerlind K. C. (2003). Physical activity and cancer Prevention???Mechanisms. Med. Sci. Sports Exerc. 35, 1834–1840. 10.1249/01.MSS.0000093619.37805.B7 14600547

[B71] Xiong X. Liu X. Li H. He H. Sun Y. Zhao Y. (2018). Ribosomal protein S27-like regulates autophagy via the β-TrCP-DEPTOR-mTORC1 axis. Cell Death Dis. 9, 1131–1142. 10.1038/s41419-018-1168-7 30425236 PMC6234217

[B72] Zhao Y. Xiong X. Sun Y. (2011). DEPTOR, an mTOR inhibitor, is a physiological substrate of SCFβTrCP E3 ubiquitin ligase and regulates survival and autophagy. Mol. Cell 44, 304–316. 10.1016/j.molcel.2011.08.029 22017876 PMC3216641

[B73] Zoncu R. Efeyan A. Sabatini D. M. (2011). mTOR: from growth signal integration to cancer, diabetes and ageing. Nat. Rev. Mol. Cell Biol. 12, 21–35. 10.1038/nrm3025 21157483 PMC3390257

